# RiFNet: Automated rib fracture detection in postmortem computed tomography

**DOI:** 10.1007/s12024-021-00431-8

**Published:** 2021-10-28

**Authors:** Victor Ibanez, Samuel Gunz, Svenja Erne, Eric J. Rawdon, Garyfalia Ampanozi, Sabine Franckenberg, Till Sieberth, Raffael Affolter, Lars C. Ebert, Akos Dobay

**Affiliations:** 1 Zurich Institute of Forensic Medicine, University of Zurich, Winterthurerstrasse 190/52, CH-8057 Zurich, Switzerland; 2 Department of Mathematics, University of St. Thomas, St. Paul, Minnesota, 55105-1079 USA; 3 Institute of Diagnostic and Interventional Radiology, University Hospital Zurich, Rämistrasse 100, 8091 Zurich, Switzerland

**Keywords:** Deep learning, Convolutional neural networks, Computed tomography, Forensic sciences, PMCT

## Abstract

Imaging techniques are widely used for medical diagnostics. In some cases, a lack of medical practitioners who can manually analyze the images can lead to a bottleneck. Consequently, we developed a custom-made convolutional neural network (RiFNet = **Ri**b **F**racture **Net**work) that can detect rib fractures in postmortem computed tomography. In a retrospective cohort study, we retrieved PMCT data from 195 postmortem cases with rib fractures from July 2017 to April 2018 from our database. The computed tomography data were prepared using a plugin in the commercial imaging software Syngo.via whereby the rib cage was unfolded on a single-in-plane image reformation. Out of the 195 cases, a total of 585 images were extracted and divided into two groups labeled “with” and “without” fractures. These two groups were subsequently divided into training, validation, and test datasets to assess the performance of RiFNet. In addition, we explored the possibility of applying transfer learning techniques on our dataset by choosing two independent noncommercial off-the-shelf convolutional neural network architectures (ResNet50 V2 and Inception V3) and compared the performances of those two with RiFNet. When using pre-trained convolutional neural networks, we achieved an F_1_ score of 0.64 with Inception V3 and an F_1_ score of 0.61 with ResNet50 V2. We obtained an average F_1_ score of 0.91 ± 0.04 with RiFNet. RiFNet is efficient in detecting rib fractures on postmortem computed tomography. Transfer learning techniques are not necessarily well adapted to make classifications in postmortem computed tomography.

**Supplementary Information:**

The online version contains supplementary material available at 10.1007/s12024-021-00431-8.

## Introduction

Deep learning (DL) describes a type of machine learning technique mostly used for image classification. Although the technique has existed since the 1980s [1–3], it was only in the last decade that deep learning has attracted more widespread attention. This renewed interest in deep learning originates from the evolution of graphics processing units (GPUs) and their ability to process heavy calculations. Today, most of the deep learning open-source frameworks take advantage of GPU acceleration for image analysis. Coupled with the burgeoning quantity of data collected through online service providers, deep learning is now widely used in a variety of applications that range from improving user experience to improving medical diagnosis to guiding self-driving vehicles.

DL techniques applied to image analysis consist of convolutional neural networks (CNNs). Features such as edges and ridges are extracted from the data using mathematical filters with various structures. The features are stored in a series of abstract images called feature maps, which are no different from a tensor in their representation. Such a tensor constitutes the basis for the input layer of a fully connected neural network by converting the tensor into a one-dimensional array or vector.

Developments with CNNs have already been made in computed tomography (CT)-based volume measurements of pericardial effusion [4], automated segmentation for CT volumes of livers [5], and automated tumor volumetry in brain tumors [6–8]. Two recent reviews on applications of machine learning [9] and deep learning [10] in postmortem forensic radiology constitute a comprehensive reference on the topic.

Rib fractures resulting from blunt force trauma or chest compression due to resuscitation attempts are common and important findings in forensic case assessment. The automation of rib fracture detection on CT images can greatly facilitate the work of forensic radiologists [11], whose ability to detect these fractures may be compromised due to the extremely high volumes of images they need to process in a day [12, 13]. In particular, an autopsy might fail to detect, for example, incomplete rib fractures, making postmortem computed tomography (PMCT) even more valuable [11]. Various groups have applied CNNs to automate fracture detection, including their localization in some cases, on X-ray radiographs, magnetic resonance imaging (MRI) and CT imaging [14–24]. Open-source projects focusing on CNNs such as Inception or ResNet constitute an attractive solution for researchers to implement custom-built models based on existing CNNs that have been previously trained on a large set of images found on the Internet. This is particularly the case in radiology and medical imaging. For instance, Mawatari et al. retrained GoogLeNet to detect hip fractures on pelvic radiographs [17]. In a recent study by Weikert et al., the authors trained a CNN based on the ResNet architecture to detect rib fractures on trauma CT scans [20]. In contrast, Jin et al. [21] decided to introduce a volumetric CNN by customizing U-Net [25], and Hu et al. combined a 2D with a 3D CNN [24]. However, all these studies have been realized with clinical CT data.

**Fig. 1:**
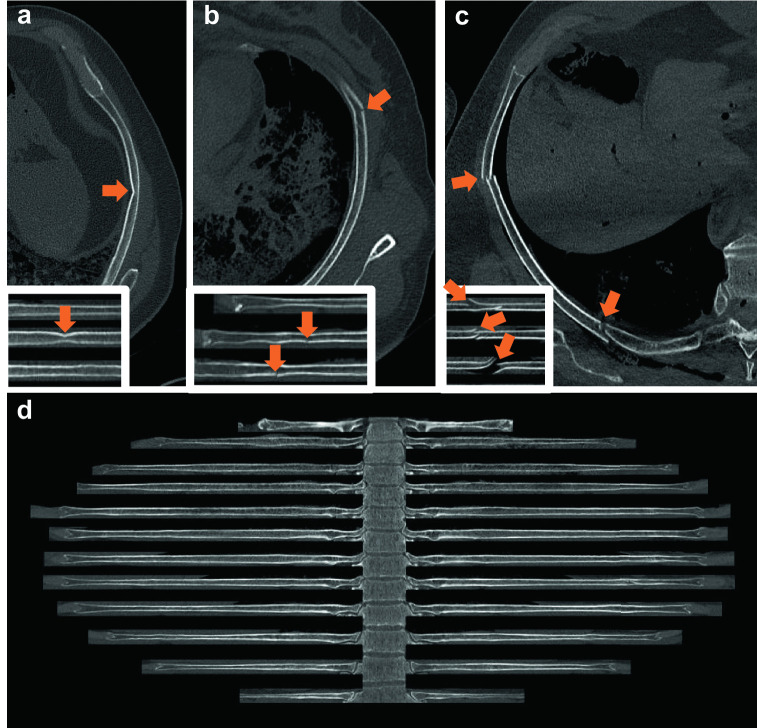
Rib unfolding. **(a)** Multi-planar view of a so-called buckle rib fracture (subgroup of incomplete rib fractures). Inset lower left: Rib unfolding view of a buckle rib fracture with no interrupted cortical line but obvious kinking. **(b)** Multi-planar view of an incomplete rib fracture; outer cortical line interrupted. Inset lower left: Rib unfolding view of an incomplete rib fracture with interrupted cortical line. **(c)** Multi-planar view of two complete rib fractures in the middle and the back of the rib. Inset lower left: Rib unfolding view of three complete rib fractures. In contrast to the two other fractures (a and b), both cortical lines are interrupted in complete fractures. **(d)** Rib unfolding with no rib fractures and 12 ribs on either side of the vertebral column. The cross-sectional images (**a**, **b**, and **c**) were extracted with bone window settings (center 450 HU, width 1,500 HU), while the window settings used for the unfolded rib images (**d**) and the insets (**a**, **b**, and **c**) were 1,000 HU for the center and 2,500 HU for the width

Among incomplete rib fractures, it is particularly important to correctly identify so-called “buckle rib fractures”, which are typically symmetrically distributed fractures along the midclavicular lines, commonly observed after cardiopulmonary resuscitation (see Fig. 1a, b, and c for more details) [26]. Thus, the correct detection and classification of rib fractures by a machine learning algorithm can be of both clinical and forensic relevance [27, 28].

CT bone reading is a tool available in the commercial imaging software Syngo.via [29]. The tool can unfold volumetric rib CT data by representing a single-in-plane image reformation of the rib cage. A spider-like image is produced with the vertebral column in the middle, with the 12 ribs distributed on each side. The ribs can be rotated around their transversal axis for interpretation. Fractures on one or multiple ribs can therefore be easily visualized on a single image (see Fig. 1d for more details).

The transfer learning technique consists of taking a validated architecture and deploying it to solve another classification problem. With this technique only the last layer (output layer) is modified to match the actual number and type of categories present in the new study. While using transfer learning on our single unfolded reformatted images to detect rib fractures we encountered several limitations. We address them in the Materials and Methods (see the transfer learning section). Due to those limitations, we decided to develop a new architecture.

**Fig. 2:**
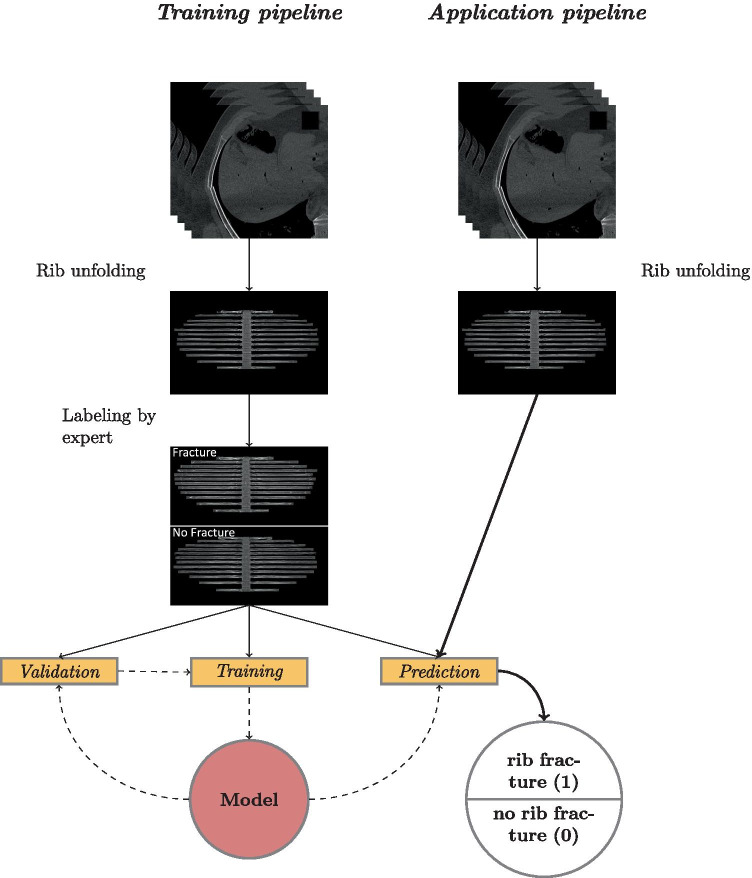
Workflow of rib fracture detections. Volumetric PMCT data of rib fractures are unfolded to form a single-in-plane image. These images are used to train a CNN (training pipeline). The pretrained CNN can be applied to detect rib fractures on single-in-plane PMCT image (application pipeline)

In this paper, we introduce a specifically designed CNN architecture, called RiFNet (**Ri**b **F**racture **Net**work), to improve rib fracture detection in single unfolded reformatted images (see Fig. 2 for a general workflow). To train, validate, and test RiFNet, we performed a retrospective cohort study. We selected a total of 195 cases from July 2017 up to April 2018 from our database. These cases served as a proxy to generate 585 postmortem computed tomography (PMCT) images with and without rib fractures. In comparison, Weikert et al. trained their model on 511 CT images collected between January and December 2018 [20], while Jin et al. collected 7,473 annotated rib fractures from 900 patients during the same period [21]. The performance of our classifier is presented in the Results section. We also compared the performance of our in-house architecture to two standard open-source off-the-shelf CNN architectures. For this purpose, we chose a residual learning network (ResNet50 V2) [30] and an improved version of Inception (Inception V3) [31].

## Materials and methods

### Ethics

The data used in this retrospective cohort study (retrospective evaluation of medicolegal scan data with new methods, publication in anonymized form) are in accordance with Swiss laws and ethical standards. Ethics approval for this study was waived by the Ethics Committee of the Canton of Zurich (KEK ZH-No. 15–0686).

### Case selection

A total of 340 consecutive postmortem cases were retrospectively retrieved from July 2017 up to April 2018 from our database. We excluded (A) cases with signs of advanced decomposition (based on the radiological alteration index (RA-Index) defined by Egger et al*.* [32]), (B) cases with organ explantation, (C) cases of severe trauma with extensive damage to the corpse such as amputation or exenteration, (D) cases with deviating scanning protocol, (E) cases without PMCT data, (F) cases in which the rib fracture was present in the volumetric CT data and the autopsy, but not visible in the rib unfolding tool or in which the rib defect was in the cartilaginous part of the rib, and (G) cases that are still under investigation. One case was excluded due to a gunshot defect in one of the accessory ribs, which could not be displayed in the rib unfolding tool. Applying the exclusion criteria, a total of 195 remaining cases were included (55 females with a median age of 64 and 140 males with a median age of 54). All these cases underwent whole-body native PMCT and were subjected to standard forensic autopsy. In our final study group, 85 cases showed only acute rib fractures, 84 had no rib fractures, and 26 had old fractures, with or without acute fractures. Both complete and incomplete rib fractures were included, independent of their location. This information was directly derived from the autopsy reports.

### Image treatment prior to classification

The value of a single voxel on PMCT images corresponds to the X-ray attenuation relative to water, given in Hounsfield units (HU). Each pixel has a value depth of 12 bits with values ranging from -1,000 (X-ray density of air) to 3,096 HU, with 0 being the X-ray density of water. Extracted images were encoded at a value depth of 8 bits. The technical parameters of the CT scans can be found in Flach et al*.* [33]. The rib fracture images were extracted from volumetric CT data (see Fig. 1a-d for more details) using the Syngo.via rib unfolding tool CT Bone Reading (Siemens Healthineers GmbH, Erlangen, Germany) with bone window settings (center 450 HU, width 1,500 HU) for the cross-sectional images. The window settings for the unfolding tool were 1,000 HU for the center and 2,500 HU for the width.

### Data mining

Imaging findings were used to label each case as either with or without rib fractures. The readout of the CT data and the labeling were performed by a medical student under the supervision of a board-certified forensic pathologist with nine years of forensic imaging experience. The data were retrieved from the multimodality image reading software Syngo.via. Each dataset represented the whole rib cage that was unfolded using the rib unfolding tool CT Bone Reading. In some cases, the reconstruction was insufficient, e.g. following mis-segmentation. Nevertheless, we included these cases as artifacts that have a distinct appearance compared to rib fractures. These images were labeled accordingly depending on whether mis-segmentation occurred on a rib cage with fractures or without fractures. Ringl et al*.* [29] also observed similar cases of faulty recognition and attempted to correct the segmentation with more than four affected ribs per image. We exported three images for each of the 195 cases, with random axial rib rotations (rotation about the vertical axis) to generate more diversity (data augmentation), and all graphics and lines hidden. The pictures were stored as portable graphic (PNG) documents with a resolution of 500 × 1,000 pixels. Finally, a total of 255 images with new rib fractures, 252 images without rib fractures, and 78 images with old rib fractures with or without new fractures were collected and used for performance testing.

### Model architecture

**Fig. 3:**
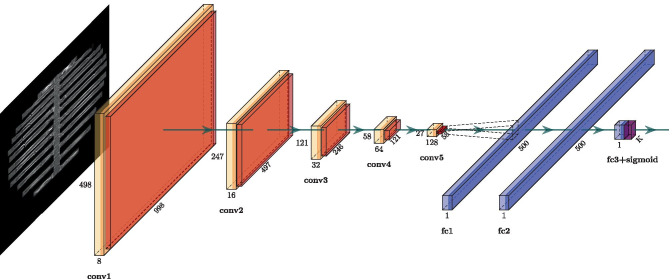
The RiFNet architecture. The architecture comprises five convolution (conv) layers, each with a max pooling layer, and a fully connected (fc) neural network consisting of two dense layers each with 500 nodes. The output layer is combined with a sigmoid function (sigmoid) for the classification (K)

Our model was developed in Python using the high-level machine learning API Keras (https://keras.io) based on the GPU version of TensorFlow version 2.0 [34]. The model was developed in close collaboration with the team responsible for the data mining (see section data mining above). The first building block of the RiFNet architecture is comprised of five convolution layers, each with a max-pooling layer. The number of output filters doubles for each convolution layer from eight to a final number of 128 output filters. For the convolution operation, we used a kernel size of 3 × 3, followed by a max-pooling operation with a kernel size of 2 × 2. A flattened layer serves as the transition from the first to the second building block. The second building block consists of a fully connected neural network composed of two dense layers with 500 nodes each, a dropout layer with a dropout rate of 0.5, and an output layer. Figure 3 shows the RiFNet architecture. To introduce nonlinearity, we used a rectified linear unit (ReLU) activation function for all layers, except the final layer, where we used a sigmoid activation function to generate binary output indicating the presence or absence of a fracture. Hence, the current version of our model does not count the number of fractures, nor identify their location. We used the adaptive moment estimation (Adam) algorithm for optimization and the binary cross-entropy loss function. The learning rate was set to 0.00015, and the batch size was set to 15. Our model was trained over 30 epochs. The architectures were trained and tested on an NVIDIA RTX 2080 Titan with 11 GB GDDR6 memory.

### Transfer learning

As already mentioned in the introduction, transfer learning describes the process of training a custom dataset on a pretrained network. Several approaches exist. The most common process is to retrain the output layer while keeping all the hidden layers frozen. Another option is to specifically train some of the hidden layers. If the computing resources are sufficient, there is the option to completely retrain a pretrained network by unfreezing the whole network and readjusting the weights of the nodes to match the custom dataset more specifically. Here, we applied this technique to compare RiFNet with two standard architectures: Inception V3 [31] and ResNet50 V2 [30]. These two architectures were pretrained on a massive dataset called ImageNet [35] inside TensorFlow and Keras. First, we trained the networks on the original size of our images (500 × 1,000) and then on the downscaled version using OpenCV with a cubic interpolation function (224 × 224 for ResNet50 V2 and 299 × 299 for Inception V3) with all the above-mentioned approaches. These two networks were trained multiple times with a learning rate ranging from 0.001 to 0.000001 and a batch size ranging from 10 to 20 for up to 300 epochs in each training attempt. However, due to the early stopping function implemented in Keras, most of our training sessions stopped before reaching 300 epochs. Inception V3 was compiled with the RMSprop optimizer, while ResNet50 V2 was compiled with the Adam optimizer. Additionally, we added two to four dense layers with 500 nodes each, and a dropout layer with a rate of 0.2 to 0.6.

### Training, validation, and prediction

**Fig. 4:**
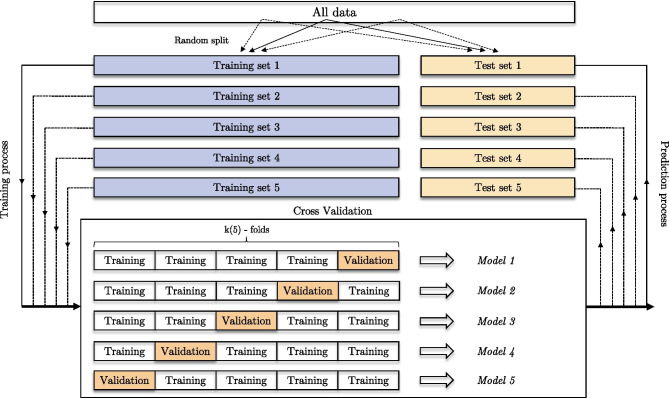
The cross-validation process. A total of 25 independent training sessions were computed using the same dataset to assess the robustness of the RiFNet architecture. This operation was performed by first splitting the whole data into training/validation datasets on one side and testing dataset on the other side (training and testing set labelled 1 to 5). Subsequently, for each training process, the images have been reshuffled in a fivefold cross-validation process. The performance of the architecture was computed by averaging all 25 runs

For training, validation, and prediction, we selected 252 CT images with no fractures and 255 with fractures. We assessed the robustness of our architecture using a cross-validation approach (Fig. 4). First, we split our data into test data (15%, 77 images) and training/validation data (85%, 430 images). Then, we split the training/validation dataset into five equal parts (folds) with the stratified K-fold function from Keras, where four folds (344 images) represent the training dataset and one-fold (86 images) represents the validation dataset. Both steps were repeated five times, resulting in a total of 25 training, validation, and prediction processes. For MobileNet, we computed the recall, precision and F_1_ score using the confusion matrix. For RiFNet, we calculated the mean values of the recall, the precision and the F_1_ score over the whole iteration process, which can be summarized by the following equations:

$$\begin{array}{cc}& \mathrm{R}=\frac{1}{\mathrm{m}\cdot \mathrm{n}}\sum_{\mathrm{i}=1}^{\mathrm{m}}\sum_{\mathrm{j}=1}^{\mathrm{n}}{\mathrm{R}}_{\mathrm{ij}}\\ & \mathrm{P}=\frac{1}{\mathrm{m}\cdot \mathrm{n}}\sum_{\mathrm{i}=1}^{\mathrm{m}}\sum_{\mathrm{j}=1}^{\mathrm{n}}{\mathrm{P}}_{\mathrm{ij}}\\ & {\mathrm{F}}_{1}=\frac{1}{\mathrm{m}\cdot \mathrm{n}}\sum_{\mathrm{i}=1}^{\mathrm{m}}\sum_{\mathrm{j}=1}^{\mathrm{n}}{\mathrm{F}}_{1}^{\mathrm{ij}}\end{array}$$

where m denotes the number of training sessions and n denotes the number of cross-validations.

## Results

**Fig. 5:**
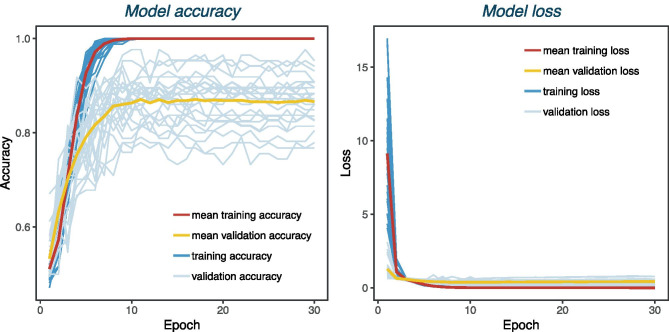
Left: Model accuracy over 30 epochs. For each of the 25 training sessions the training and validation accuracy is plotted, as well as the mean training (max. 1.0) and validation accuracy (max. 0.87). Right: Model loss over 30 epochs. Development of training and validation loss with mean values (min. 0, min. 0.4 respectively) for 25 training sessions

**Fig. 6:**
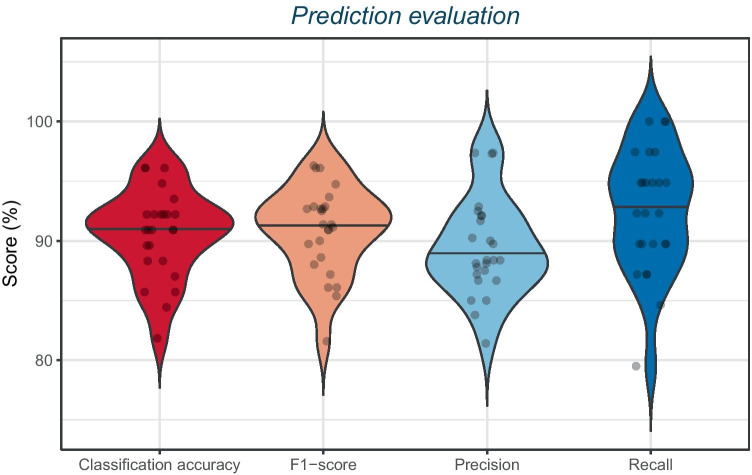
Evaluation of 25 predictions on unseen data viewed as violin plots. RiFNet achieved an overall classification accuracy mean value of 91 ± 4%, a mean F_1_ score value of 91 ± 4%, a mean precision value of 89 ± 3%, and a mean recall value of 93 ± 5%

In a first attempt, we relied on the idea that transfer learning using large pretrained CNNs such as Inception V3 or ResNet50 V2 would be a good strategy. However, this operation requires rescaling the images to the size used to train these CNNs. Training Inception V3 and ResNet50 V2 on our original image size (500 $\times$ 1,000) did not work due to a lack of computational power. When we downscaled the images (224 × 224 for ResNet50 V2 and 299 × 299 for Inception V3) and retrained the output layer by keeping the hidden layers unchanged, the validation accuracy remained at approximately 0.5 for all possible combinations of parameters described in the transfer learning section. After allowing the weights of the architecture to be adjusted, we noticed that the networks quickly overfitted our data even before the validation accuracy started to increase. Therefore, by adjusting the weights only on the batch normalization layers, we achieved a recall on unseen test data of 0.63 and 0.61, a precision of 0.65 and 0.62 and an F_1_ score of 0.64 and 0.61 for Inception V3 and ResNet50 V2, respectively. With RiFNet, we achieved an overall mean recall of 0.93 ± 0.05 and a mean precision of 0.89 ± 0.03, leading to a mean F_1_ score of 0.91 ± 0.04. Figure 5 shows the training progress, and Fig. 6 the prediction evaluation of RiFNet using violin plots. Table 1 gives an overview of the overall performance analysis. We also tested whether downscaling the images can potentially cause a loss of features (e.g. rib fractures). For this part, we repeated the cross-validation process and trained RiFNet with a lower image size (224 × 224). We found that the mean F_1_ score dropped by 14% (0.77 ± 0.06).

**Table 1:** Performance analysis overview

Model	Image size (pixels)	Recall	Precision	${\mathrm{F}}_{1}$ Score
Inception V3	299 $\times$ 299	0.63	0.65	0.64
ResNet50 V2	224 $\times$ 224	0.61	0.62	0.61
RiFNet (mean)	500 $\times$ 1000	0.93 ± 0.05	0.89 ± 0.03	0.91 ± 0.04

## Discussion

Detection of rib fractures in PMCT is important not only in clinical but also in forensic imaging. We developed a tool based on a CNN architecture that can detect rib fractures on PMCT images. These images were obtained using the rib unfolding tool CT Bone Reading in Syngo.via. The authors who developed the tool reported a higher reader performance and overall sensitivity when using the tool (81.1%) compared to the standard (80.3%) [29]. Based on these results, we estimated that the information on the rib fractures contained in a single-in-plane image reformation of the rib cage is comparable to the standard transverse, coronal, and sagittal orientations. By using RiFNet, we correctly classified 89 out of 100 rib fractures on average, and only five to 13 out of 100 images were incorrectly classified (see Supplementary Fig. S1 for false positive and false negative examples). These results were better than those obtained with two standard open-source off-the-shelf CNN architectures. The discrepancy between Inception V3 and ResNet50 V2 on one side and RiFNet on the other side can be partially explained by the fact that accessible pretrained CNNs are mainly trained on smaller image sizes. By downscaling our images, we most likely lost information about the features we were interested in. Furthermore, our classification task was entirely different from the task pretrained networks were trained on (see Transfer learning section). In contrast, our network is specifically adjusted to these circumstances. The current version of TensorFlow 2.0 arguably gives the option to retrain all the weights in any of the supplied architectures with pretrained weights. However, this operation required more computing power and was out of reach in our case, as stated above in the Results section. Having such a gap in computing power between deploying a complex pretrained CNN and developing a custom solution that is simpler in its architecture but adapted to the problem is potentially more attractive for forensic pathologists working with PMCT images.

As mentioned previously, “buckle rib fractures”, as a subgroup of incomplete fractures, are of forensic relevance. RiFNet can detect rib fractures on PMCT images with a mean F_1_ score of 0.91 ± 0.04%. However, we did not train RiFNet to discriminate between complete and incomplete fractures. For this part, we would need to train RiFNet on a larger dataset containing more examples of complete and incomplete rib fractures. With such a dataset, a classifier could be built to sort PMCT images into three different classes: those containing no fractures, those with incomplete fractures, and those with complete fractures. The current version of RiFNet cannot count the number of distinct fractures present in an image. This feature would require embedding an image segmentation algorithm such as those based on encoder-decoder networks to identify the position of the features that have been extracted. However, this information is less instrumental than missing a fracture. We also did not test the effect of aging and whether RiFNet would perform differently in specific age groups.

Understanding and interpreting medical images requires special training. Even with an expert eye, interpreting medical images can be difficult. It is not surprising for learning algorithms to encounter difficulties in classifying medical images. Achieving a mean F_1_ score of 0.91 ± 0.04% indicates that only 10% of the images are misclassified. Methods to reduce the number of false positives warrant further investigation.

## Conclusion

The use of deep learning techniques in medical imaging has gained much momentum in recent years. The constant development of existing frameworks such as TensorFlow, PyTorch, or Keras provides the scientific community with a series of pretrained CNNs. With our current study, we concluded that these pretrained CNNs are not necessarily adaptable for all types of problems encountered in medical imaging, especially with the whole-body PMCT imaging, where it is common to work with large datasets with high resolution to ensure that every anomaly can be detected. Retraining existing CNNs is only possible with substantially more powerful computing resources than those required to deploy the pretrained versions. We addressed this gap by developing a custom-made solution for PMCT imaging.

## Key points

1. RiFNet addresses a gap to facilitate the work of forensic radiologists.
2. RiFNet can achieve a classification accuracy of 91%.
3. RiFNet is an easily adaptable solution for postmortem computed tomography images.
4. RiFNet outperforms pretrained ResNet50 V2 and Inception V3.

## Supplementary Information

Below is the link to the electronic supplementary material.

Supplementary file1 (DOCX 459 KB)

## Data Availability

The source code of the model can be made available upon request.
