# Supplementary material for: RiFNet: Automated rib fracture detection in postmortem computed tomography
Source: Forensic Sci Med Pathol. 2021 Oct 28;18(1):20–9. doi: 10.1007/s12024-021-00431-8 (PMC8921053; doi:10.1007/s12024-021-00431-8)

**Fig. S1** Four examples of misclassified single-in-plane PMCT image reformation of the rib cage. Two false positives (upper row) where RiFNet falsely detected the presence of rib fractures, and two false negatives (lower row) where RiFNet did not detect the presence of rib fractures indicated by the arrows. Bone window settings: center 1,000 HU, width 2,500 HU.


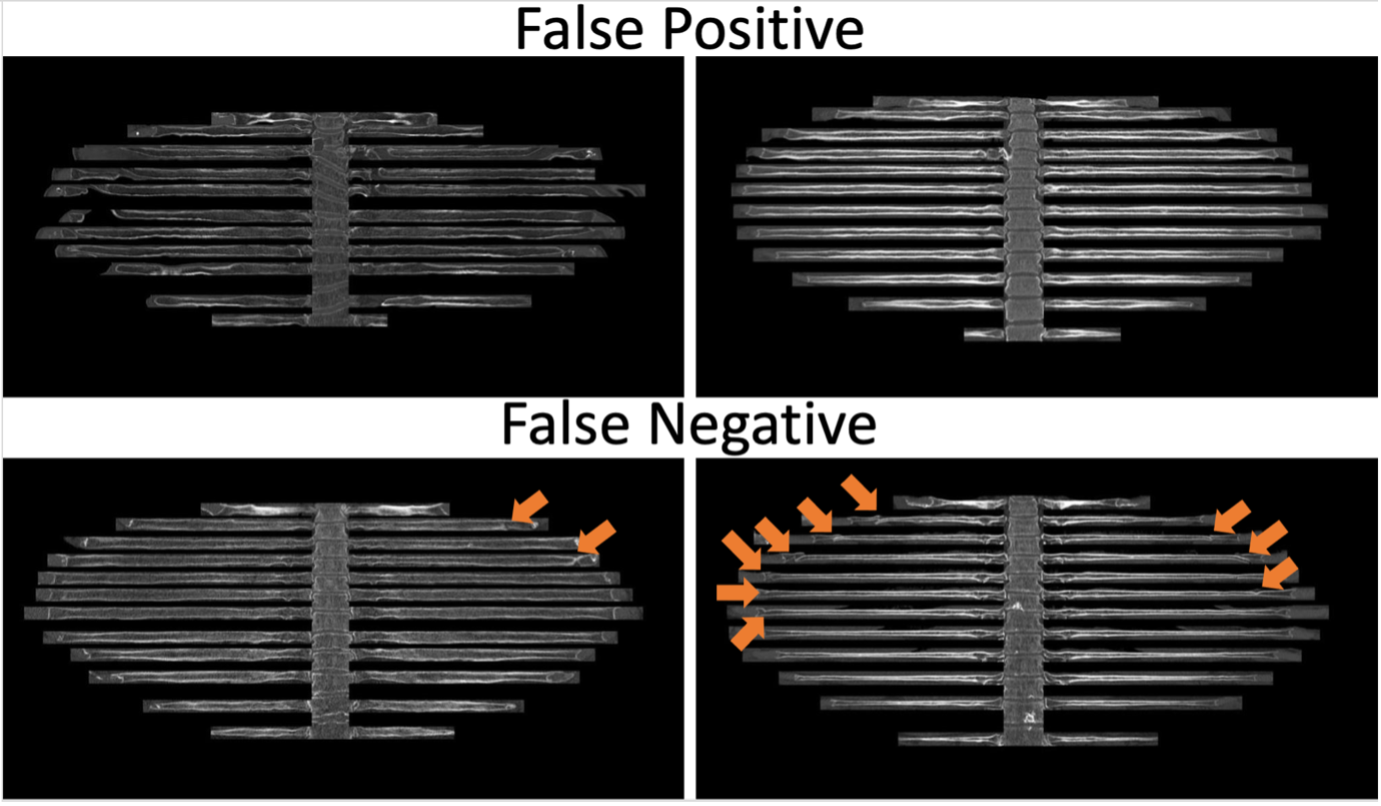

Supplement: Supplementary file 1 — Supplementary file1 (DOCX 459 KB) [file 12024_2021_431_MOESM1_ESM.docx]
